# A Pediatric Case of Relapsing-Remitting Multiple Sclerosis Onset following Varicella Zoster Ophthalmicus with Optic Neuritis

**DOI:** 10.1155/2018/6931206

**Published:** 2018-03-26

**Authors:** Naoko Shiba, Yuji Inaba, Mitsuo Motobayashi, Makoto Nishioka, Yoichiro Kawasaki, Shunsuke Noda, Hiroki Matsuura, Norimoto Kobayashi, Takafumi Matsuoka, Akinori Nakamura, Yozo Nakazawa

**Affiliations:** ^1^Department of Pediatrics, Shinshu University School of Medicine, Matsumoto, Japan; ^2^Regenerative Science and Medicine, Shinshu University, Matsumoto, Japan; ^3^Division of Neurology, Nagano Children's Hospital, Azumino, Japan; ^4^Matsuoka Pediatric Clinic, Matsumoto, Japan; ^5^Intractable Disease Care Center, Shinshu University Hospital, Matsumoto, Japan

## Abstract

Some epidemiological studies have implied a pathogenetic association between varicella zoster virus (VZV) and multiple sclerosis (MS); this, however, remains controversial. The present report describes a case involving an immunocompetent 10-year-old girl who developed relapsing-remitting MS following the prolonged reactivation of VZV inside the first branch of the trigeminal nerve, exhibiting herpes zoster ophthalmicus with severe optic neuritis. Symptoms related to herpes zoster ophthalmicus and MS appeared consecutively in the 10-week period after the appearance of vesicles. This suggests that the onset of MS was triggered by some mechanism involving VZV reactivation in the first branch of the trigeminal nerve. To the best of our knowledge, this report is the first to describe a relationship between the onset of MS and herpes zoster ophthalmicus. Early diagnosis and aggressive antiviral therapy are important in cases of herpes zoster ophthalmicus to prevent the possible development of MS as well as visual impairment as sequela.

## 1. Introduction

Varicella zoster virus (VZV) is a strongly neurotropic virus that has been hypothetically associated with multiple sclerosis (MS). A recent large-cohort epidemiological study identified a higher risk for MS development in patients who experienced a herpes zoster (HZ) attack within the previous year than in control subjects [1]. Some studies have reported the presence of VZV DNA in mononucleocytes and the cerebrospinal fluid (CSF) of patients during MS onset or relapse, suggesting a direct role for VZV in MS pathogenesis [2, 3]. This, however, remains controversial, and to date, only a few case reports proposing a causal relationship between VZV infection and MS onset have been published [4]. In this report, we present a pediatric case of relapsing-remitting MS (RRMS) directly following HZ ophthalmicus (HZO) caused by the reactivation of VZV inside the ophthalmic branch of the trigeminal nerve.

## 2. Case Presentation

**Figure 1:**
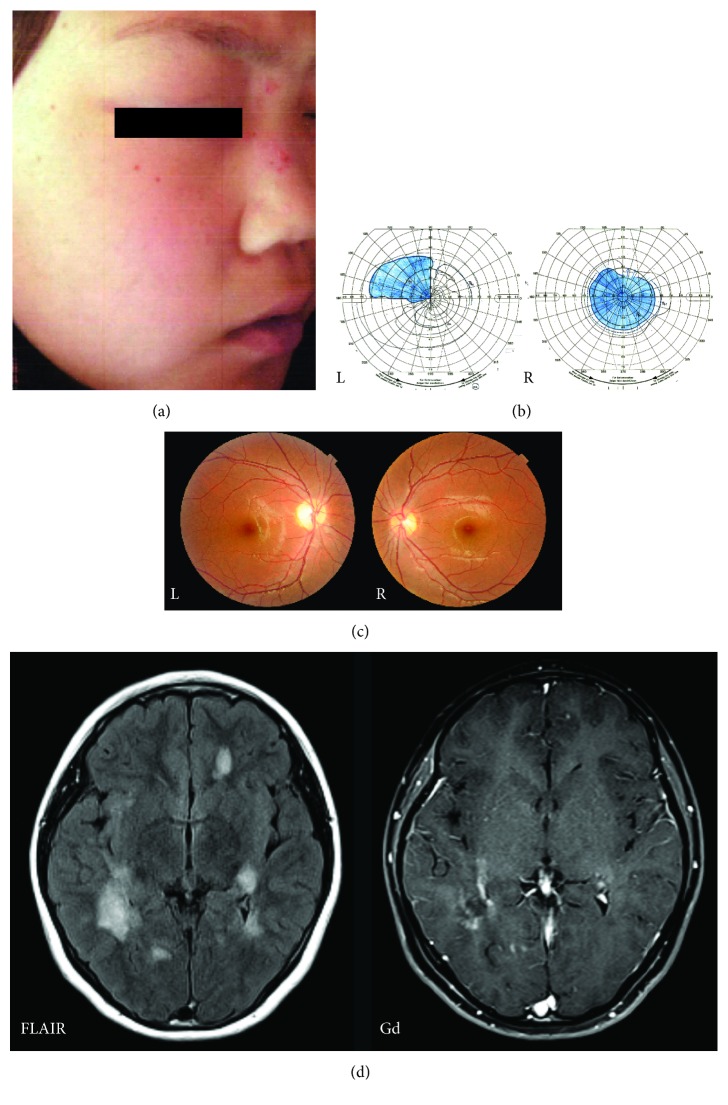
Clinical findings. (a) Skin rashes over the nasal sidewall and peripheral facial palsy on the right side 3 months before admission. (b) A visual field test on admission identified a central scotoma in the right eye and quadrantanopia in the left (L) eye. (c) The optic disc was atrophied and pale in the right (R) eye; there were no abnormal findings in the left eye. (d) Brain magnetic resonance imaging (MRI) on admission. Fluid-attenuated inversion recovery (FLAIR; left) imaging demonstrated multiple hyperintense periventricular deep white matter and juxtacortical lesions, some of which exhibited an open ring pattern on T1-weighted imaging after gadolinium (Gd) enhancement (right).

**Figure 2:**
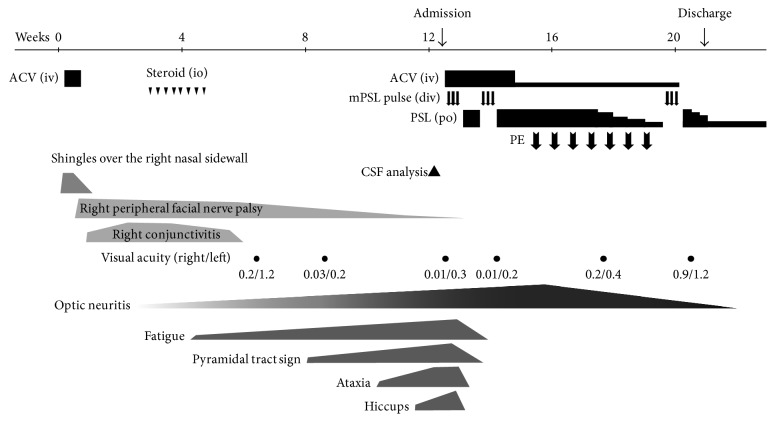
Clinical course of HZ ophthalmicus and multiple sclerosis at onset. ACV, acyclovir; CSF, cerebrospinal fluid; mPSL pulse, methylprednisolone pulse therapy; PE, plasmapheresis; po, per oral administration; io, intraocular administration; iv, intravenous administration.

A previously healthy 10-year-old girl with a history of chicken pox at 4 months of age presented with HZ over the right side of the nasal sidewall (Figures 1(a) and 2), accompanied by right-sided peripheral facial palsy, ophthalmic pain, lacrimation, and headache. No abnormal findings were identified on initial brain magnetic resonance imaging. A five-day course of intravenous acyclovir improved the skin lesions; however, the ophthalmic pain worsened, and she was diagnosed with conjunctivitis 2 weeks later. Despite topical steroid therapy, severe ophthalmoplegia persisted for one month. As the pain was relieved, she noticed a vision decrease (0.2/1.2 (right/left)), and visual acuity degraded over the following week to involve the other side. She gradually developed fatigue, muscle weakness, and numbness in the extremities. Ten weeks after the emergence of shingles, the patient began to experience dizziness and continuous hiccups when she was referred to the authors' clinic. A neurological evaluation revealed bilateral pyramidal tract signs, cerebellar ataxia, and severe visual impairment (0.01/0.3), with a central scotoma in the right eye (Figure 1(b)). The right optic disc was pale and slightly atrophic (Figure 1(c)). Brain magnetic resonance imaging revealed multiple high-intensity lesions involving the brainstem, some of which had an open ring or a nodular pattern on gadolinium enhancement (Figure 1(d)). CSF analysis at the time of hospitalization, 12 weeks after the appearance of HZ and approximately 4 weeks after the start of development of the brain lesions (Figure 2), revealed elevated myelin basic protein level (377 ng/L (normal, <102 ng/L)) and the presence of oligoclonal bands, with normal total protein level (37 mg/dl (normal, 15–45 mg/dl)) and no pleocytosis. VZV- and rubella-specific IgG indices were elevated (4.2 and 4.9, resp. (normal <1.5)), which implicated the intrathecal polyspecific humoral immune response common in MS [5]. Results of polymerase chain reaction tests for VZV DNA in the CSF and peripheral mononucleocytes at the same time point were negative, and anti-aquaporin-4 and myelin oligodendrocyte glycoprotein antibodies were not detected in the serum. A diagnosis of MS was made according to the revised McDonald criteria [6], and the patient was administered high-dose methylprednisolone pulse therapy (25 mg/kg/day) three times per week for two consecutive weeks and a simultaneous two-week course of intravenous acyclovir (80 mg/kg/day). Brain lesions and focal neurological signs improved dramatically in response to this therapy; however, the optic neuritis was steroid resistant. It finally responded to second-line treatment with plasmapheresis (seven cycles), and visual acuity improved gradually and finally recovered to 1.2 on both sides two months later. Under oral corticosteroid therapy, the patient experienced first relapse with multiple gadolinium-enhanced white matter lesions in the cerebrum 11 months after initial onset. At this point, no symptoms of VZV reactivation were present, and IgG indices for VZV and rubella were negative. Treatment with intramuscular interferon-beta, as a disease-modifying therapy, was initiated. Currently, 5 years after initial onset, the patient is without neurological sequelae despite multiple recurrences (10 times) of self-limited or steroid-reactive deep white matter brain lesions.

## 3. Discussion

HZO is defined as the reactivation of VZV inside the ophthalmic branch of the trigeminal nerve [7], commonly manifesting as conjunctivitis, anterior uveitis, or keratitis and rarely presenting with optic neuritis [8]. Optic neuritis in HZO usually develops within a few months after the appearance of skin lesion, sometimes involving the eyes bilaterally [8]. The precise mechanism underlying optic neuritis in HZO remains unclear, although several hypotheses have been proposed, including direct viral nerve infection through the cavernous sinus, CSF and/or transneural viral spreading, and immune-mediated inflammation. Early systemic administration of a high-dose antiviral agent is highly recommended to prevent a poor visual prognosis.

In the current case, it is possible that optic neuritis developed as a symptom of MS before the brain demyelinating lesions. However, sequential appearance of HZ, conjunctivitis, and optic neuritis in a 10-week period and the ipsilateral severity of symptoms suggested that optic neuritis developed pathologically associated with HZO. Furthermore, optic neuritis was steroid resistant, contrary to the brain lesions, and completely remitted in response to plasmapheresis. This is indicative of a mechanism different from that for brain lesions, possibly including more involvement of circulating substances in serum such as autoantibodies, immune complexes, and cytokines.

It is noteworthy that the delay in administration of antiviral therapy may have also contributed to the exacerbation of optic neuritis. Additionally, we highlight the possibility that persistent HZO with optic neuritis triggered the onset of RRMS, although it is undetermined whether VZV had invaded into the central nervous system or not prior to the demyelination, because the analysis of DNA in CSF and peripheral blood was performed 4 or more weeks later than the appearance of the focal neurological symptoms, which is somewhat late for evaluation of the presence or absence of infection. It has been recently reported that pain-mediated neural signals could induce an immunological reaction that triggered relapse in a mouse model of MS, which is termed the “pain gateway reflex” [9]. In the current case, severe pain associated with HZO may have induced some type of gateway reflex, which may, at least, be a partial triggering factor for the onset of optic neuritis or MS.

To the best of our knowledge, this is the first case report of RRMS onset directly following HZO, with severe optic neuritis. The consecutive and immediate appearance of symptoms related to HZO and MS in a 10-week period after the onset of skin lesions strongly suggests that VZV reactivation in the first branch of the trigeminal nerve immunologically triggered the onset of MS.Ocular complications, including optic neuritis, are rare in patients with pediatric herpes zoster, and their symptoms cannot be easily noticed in children. However, early diagnosis and aggressive antiviral therapy are important in cases of HZO to prevent the possible development of MS as well as visual impairment as sequela. Further studies are needed to determine the exact role of antecedent and concomitant infection with VZV in the onset of MS.
